# Deviation of Trypsin Activity Using Peptide Conformational Imprints

**DOI:** 10.3390/nano11020334

**Published:** 2021-01-27

**Authors:** Kiran Reddy Kanubaddi, Pei-Yu Huang, Ya-Lin Chang, Cheng Hsin Wu, Wei Li, Ranjith Kumar Kankala, Dar-Fu Tai, Chia-Hung Lee

**Affiliations:** 1 Department of Life Science, National Dong Hwa University, Hualien 97401, Taiwan; 810513107@gms.ndhu.edu.tw (K.R.K.); ranjithkankala@hqu.edu.cn (R.K.K.); 2 Department of Chemistry, National Dong Hwa University, Hualien 97401, Taiwan; 610012029@gms.ndhu.edu.tw (P.-Y.H.); m9812003@ems.ndhu.edu.tw (Y.-L.C.); m9812022@gms.ndhu.edu.tw (C.H.W.); ga025361@yahoo.com.tw (W.L.); 3 College of Chemical Engineering, Huaqiao University, Xiamen 361021, China

**Keywords:** porcine pancreatic trypsin, molecularly-imprinted polymers, magnetic particles, conformational imprint, secondary structure

## Abstract

In this study, a methodology utilizing peptide conformational imprints (PCIs) as a tool to specifically immobilize porcine pancreatic alpha-trypsin (PPT) at a targeted position is demonstrated. Owing to the fabrication of segment-mediated PCIs on the magnetic particles (PCIMPs), elegant cavities complementary to the PPT structure are constructed. Based on the sequence on targeted PPT, the individual region of the enzyme is trapped with different template-derived PCIMPs to show certain types of inhibition. Upon hydrolysis, *N*-benzoyl-L-arginine ethyl ester (BAEE) is employed to assess the hydrolytic activity of PCIMPs bound to the trypsin using high-performance liquid chromatography (HPLC) analysis. Further, the kinetic data of four different PCIMPs are compared. As a result, the PCIMPs presented non-competitive inhibition toward trypsin, according to the Lineweaver-Burk plot. Further, the kinetic analysis confirmed that the best parameters of PPT/PCIMPs ^233–245+G^ were *V_max_* = 1.47 × 10^−3^ mM s^−1^, *K_m_* = 0.42 mM, *k_cat_* = 1.16 s^−1^, and *k_cat_*/*K_m_* = 2.79 mM^−1^ s^−1^. As PPT is bound tightly to the correct position, its catalytic activities could be sustained. Additionally, our findings stated that the immobilized PPT could maintain stable activity even after four successive cycles.

## 1. Introduction

Porcine pancreatic alpha-trypsin (PPT), a proteolytic enzyme, is a pancreatic serine protease (EC 3.4.21.4) with specificity for arginine or lysine substrate towards catalytic hydrolysis on esters and amides, under mild reaction conditions [1,2,3]. Owing to these facts, this proteolytic enzyme is often utilized in industrial and biomedical applications [1,4,5]. However, such enzymes are often immobilized into various substrates to improve stability and reusability without affecting their activity [2,6,7]. In this vein, various enzyme immobilization methods have been reported, such as covalent linkage, non-covalent adsorption, and encapsulation systems, among others [8,9,10,11,12]. Nevertheless, the quest for optimum performance is still on due to their conformational changes during immobilization [11,13]. Although an enzyme possesses a uniform structure, it often changes the conformation continuously. Consequently, the immobilized biocatalyst is organized randomly during/after immobilization, resulting in different constitutions and a less dynamic form.

In recent times, the utilization of molecularly imprinted polymers (MIPs) has become an emerging carrier-bound system for the immobilization of biomolecules, as they offer reversible orientation [14]. In this context, the incorporation of MIPs with magnetic particles (MPs) was first demonstrated by Ansell and Mosbach [15]. Since then, several efforts have been dedicated to the utilization of MIPs for diverse applications. In a case, Tong and colleagues applied MPs to recognize Ribonuclease A [16]. In another case, Jing and coworkers used lysozyme as a template to adsorb blood specimens with a detection limit at 5 ng/mL [17]. Previously, trypsin was utilized as the template to generate MIPs for different assays and inhibition studies [18]. Nonetheless, the employment of such a template for MPs was restricted to the usage of the whole protein.

**Figure 1:**
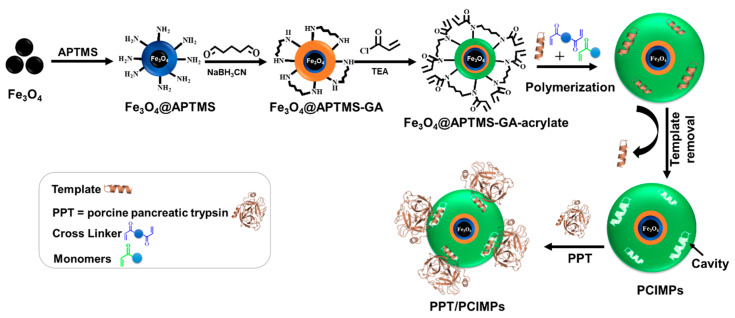
Scheme illustrating the fabrication of peptide conformational imprints (PCIs) on magnetic particles (MPs) and their binding to porcine pancreatic alpha-trypsin (PPT).

Considering these facts, herein, we demonstrate an elegant method to immobilize PPT. As segment-mediated MIPs were fabricated on MPs, cavities complementary to PPT structure were constructed on those nanomaterials. As the imprinting of a random coil peptide successfully generates the desired nano-cavity for the corresponding peptide/protein [19,20], recently, a helical peptide was utilized as a template to generate helical cavities with high affinity for the target protein, having achieved satisfactory results [21]. Accordingly, in this work, several PPT peptide segments were selected to generate helix cavities, using peptide conformational imprints decorated over the magnetic particles (PCIMPs). This study is divided into four main steps: (i) Fabricating peptide conformational imprint (PCIs) on MPs carriers; (ii) adsorbing trypsin to PCIMPs; (iii) analyzing the binding kinetics of immobilized PPT; and (iv) evaluating the reusability of immobilized PPT. The immobilization of PPT based on the PCIs was carried out as illustrated in Figure 1.

## 2. Materials and Methods

### 2.1. Reagents and Chemicals

3-(Aminopropyl) trimethoxysilane (APTMS) and ammonium acetate (NH_4_Ac) were obtained from Acros Ltd. (Fair Lawn, New Jersey, United States). Iron (III) chloride hexahydrate (FeCl_3_·6H_2_O) and triethylamine (TEA) were purchased from Merck Ltd. (Darmstadt, Germany). *N*,*N*′-Ethylene bisacrylamide (EBAA) and *N*-benzyl acrylamide (BAA) were acquired from Lancaster (Lancashire, UK). Glutaraldehyde (GA) was obtained from Ferax (Berlin, Germany). All Fmoc-amino acids were purchased from BAChem (Bubendorf, Switzerland). Acrylamide (AM), acetic acid, *N*-benzoyl-L-arginine ethyl ester (BAEE), sodium cyanoborohydride (NaBH_3_CN), *N*,*N*,*N*′,*N*′-tetramethyl ethylene diamine (TEMED), tris (hydroxymethyl) amino-methane, porcine pancreatic trypsin (PPT), Tween^®^20, and urea were acquired from Sigma Co. Ltd. (St. Louis, MO, USA). Acetone, acetonitrile (ACN), dichloromethane (DCM), *N*,*N*-dimethyl formamide (DMF), piperidine, and toluene of High-Performance Liquid Chromatography (HPLC) grade were used. Purified distilled water acquired from a Milli-Q water purification system was used in all the experiments.

### 2.2. Template Synthesis

The peptide segments, such as PPT^107–116^ (KLSSPATLNS), PPT^145–155^ (KSSGSSYPSLL), PPT^169–178^ (KSSYPGQITG), and PPT^233–245+G^ (NYVNWIQQTIAANG), were produced through the Fmoc (fluorenylmethoxycarbonyl) solid-phase peptide synthesis approach using a Discover SPPS Microwave Peptide synthesizer (Kohan Co. Ltd., Taipei, Taiwan) available at the National Dong Hwa University (Hualien, Taiwan) [22].

### 2.3. Preparation of PCIs on MPs

#### 2.3.1. Construction of Fe_3_O_4_@APTMS-GA

The synthesis of the Fe_3_O_4_ precursor, and subsequent immobilization of amine functionality, Fe_3_O_4_@APTMS, were performed as described previously [23,24]. Further, glutaraldehyde (GA) was coupled with Fe_3_O_4_@APTMS to construct stable secondary amine nanoparticles. Briefly, 100 mg of Fe_3_O_4_ @APTMS was initially placed in 50 mL of ACN and subjected to ultrasonication for 30 min. Then, 162 μL of GA was added to the mixture. Further, a few drops of acetic acid were added to maintain the weakly acidic state of the reaction mixture, and stirring was performed for 2 h. Subsequently, 200 mg of NaBH_3_CN were added, and vigorous stirring was executed for another 2 h to make the reaction mixture weakly alkaline. Finally, the resultant particles were recovered with a strong magnet, washed several times with a solvent mixture of (H_2_O:ACN = 1:1), and dried under vacuum.

#### 2.3.2. Synthesis of Fe_3_O_4_@APTMS-GA-Acrylate

To prepare Fe_3_O_4_@APTMS-GA-acrylate, 300 mg of GA-modified MPs were initially dispersed in 25 mL of dry DCM and stirred for 15 min after adding TEA (0.48 mL). Then, acryloyl chloride (0.3 mL, 3.75 mmol) was added in a drop-wise manner to the mixture at 0 °C under N_2_ purge and stirred for 24 h. Finally, the resultant product was washed with DCM and dried under vacuum.

#### 2.3.3. Preparation of PCIMPs

To prepare PCIMPs, initially, 211.2 mg of *N*, *N’*-ethylene bisacrylamide (EBAA), 56.4 mg of benzyl acrylamide (BAA), and 25.2 mg of acrylamide (AA) were dissolved in a solvent mixture containing 16 mL of PBS (pH-7.6, 20 mM) and 2 mL of ethanol. Then, 7.5 μmol of template molecules (PPT^107–116^, PPT^145–155^, PPT^169–178^, and PPT^233–245+G^) were dissolved separately in 20 mL of a solvent mixture of TFE and PBS at a ratio of 7:3 to exhibit the helical structure in the polymerization system. Further, the above two reaction mixtures were mixed after a while, and 90 mg of Fe_3_O_4_@APTMS-GA-Acrylate was added to make a pre-self-assembly reaction mixture. Then, 240 μL (10%, *w*/*w*) of ammonium persulfate and 90 μL (5%, *w*/*v*) of TEMED were added to the reaction and stirred for 24 h in the presence of N_2_ at RT. The template removal was performed based on previous studies [25,26]. According to the following articles, acetic acid as a solvent disrupts the electrostatic interactions between the template and the polymer matrix, which can be separated. Notably, the template removal process could be achieved in few minutes. Finally, the polymer-MPs were obtained and washed with 25 mM urea (aq) containing 5% acetic acid and 0.5% tween-20 to remove the template. Subsequently, the pore structures formed after the removal of the four different templates were denoted as PCIMPs^107–116^, PCIMPs^145–155^, PCIMPs^169–178^, and PCIMPs^233–245+G^, respectively.

### 2.4. Determination of Binding Affinities of PCIMPs

Notably, the binding experiments were carried out in 10 min to avoid adsorption at non-specific binding sites on PCIMPs. Briefly, 10 mg of PCIMPs was added to PBS (pH-7.6, 20 mM) containing PPT at different concentrations (0.125, 0.25, 0.5, 1, and 1.5 mg/mL) and the resulting mixture was shaken for 10 min. Then, 200 µL of supernatant was collected and measured by Fluorescence Microplate Reader at λex/λem = 290 nm/350 nm. Each experiment was repeated three times, and the results of the binding studies were evaluated using the Scatchard Equation (1) [27,28,29].

[RL]/[L] = (B_max_ − [RL])/K_d_ (1)

where [L] is the concentration of PPT in the solution, [RL] is the concentration of bound PPT, B_max_ denotes the maximum number of binding sites, and K_d_ is the dissociation constant of the ligand.

### 2.5. Activity Assay of PPT and Immobilized PPT (PPT/PCIMPs)

The catalytic activity of PPT and PPT/PCIMPs was measured using the HPLC method. *N*-benzoyl-L-arginine ethyl ester (BAEE) was utilized as the starting material, while the product was *N*-Benzoyl-L-Arginine (BA), which was observed with time. The percentage of hydrolysis rate was calculated using the following Equation (2):

(2) $$\mathrm{Hydrolysis}\;\mathrm{rate}\;\left(\%\right)=\;\frac{\mathrm{Product}\;\mathrm{area}\;\mathrm{ratio}}{\left(\mathrm{Starting}\;\mathrm{area}\;\mathrm{ratio}\;+\;\mathrm{product}\;\mathrm{area}\;\mathrm{ratio}\right)}\times 100$$

For determining the catalytic activity of PPT, initially, 1 mL mixtures possessing different BAEE concentrations (0.5, 1.0, and 1.5 mM) were prepared using 50 mM of Tris-HCl buffer, with a pH equal to 7.6. Then, 20 μL of 1mM HCl containing 30 μg PPT was formulated. The assay was started by adding 20 μL of 1 mM HCl/30 μg PPT to 1 mL mixtures with the three BAEE concentrations mentioned above, respectively. For every min, 40 μL of the solution was collected from the reaction mixture and dissolved in 500 μL of ACN: buffer = 15:85, and 99.5 μL of the resultant solution was injected for HPLC detection until the end of the reaction.

### 2.6. PPT/PCIMPs Activity Assay

Briefly, 10 mg of each PPT/PCIMPs were separately added to 8.8 mL of the BAEE solutions (0.5, 1.0, and 1.5 mM), and for every min, 80 μL of that solution were separated from the mixture and dissolved in 1 mL of ACN: buffer = 15:85. From this, 99.5 μL of the solution was collected for HPLC detection until the end of the reaction. The same procedure was also carried out for the reusability test.

An intelligent, high-performance liquid chromatography (HPLC, model L7100, Hitachi, Tokyo, Japan) set-up equipped with a UV detector (Hitachi model L-2420, Tokyo, Japan), an autosampler (Hitachi L-2200, Tokyo, Japan), and a Vercopak-RP C18 column (Vercotech Corp., Taipei, Taiwan) was used to determine the purity of peptides and for performing the kinetic analysis of the immobilized enzyme. In the hydrolysis test, the mobile phase of HPLC was composed of 0.38 mL of phosphoric acid, 0.47 mL of triethylamine, and 1 L of DI-H_2_O. The solution was then adjusted to pH 2.4 with NaOH and HCl. The ultraviolet wavelength was set at 214 nm.

### 2.7. Determination of Kinetic Constants of PPT and PPT/PCIMPs

The kinetic parameters of PPT and PPT/PCIMPs were evaluated from the Michaelis–Menten plot obtained from the following Equation (3),

(3) $$\nu =\frac{V_{max}\;\left[S\right]}{\left(K_{m}+\left[S\right]\right)\;}$$

where *v* is the reaction velocity at [S], *V_max_* is the maximum rate of the reaction, *K_m_* is the Michaelis half-saturation constant, and [S] is the concentration of the substrate.

The turnover number (*k_cat_*) was calculated using the below Equation (4).

(4) $$k_{cat}=\;V_{max}\;/\left[E\right]$$

where [*E*] is the enzyme concentration [13].

## 3. Results and Discussions

### 3.1. Rational Selection of the Template

**Figure 2:**
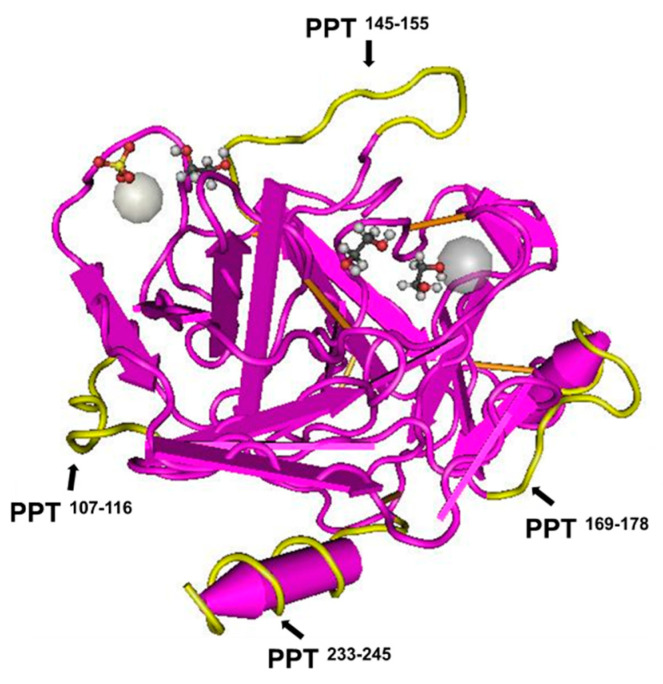
The structure of porcine pancreatic trypsin (cylinder: α-helix; arrow: β-sheet). The selected sequences are in yellow. These segments consist of the series: i.e., PPT^107–116^, PPT^145–155^, PPT^169–178^, and PPT^233–245^. The crystal structure of PPT was reproduced from http://www.ncbi.nlm.nih.gov/ and PDB ID: 1S81 [33].

The template for the imprinting was chosen considering the following parameters: (i) Peptide segments from the flank part of the PPT spatial structure were selected as the template. Due to five disulfide linkages connected among PPT, the choice of peptide segments able to influence catalysis is limited. (ii) The length of the peptide segments in the template is a significant parameter. For instance, short peptide residues form flexible structures that can help the imprinting and protein-rebinding processes [19,30]. Therefore in this study, four PPT peptides, specifically PPT^107–116^, PPT^145–155^, PPT^169–178^, and PPT^233–245^, were chosen. The locations of these segments are shown in Figure 2. At one end of the PPT^233–245^ peptide, a glycine (G) residue was added to make a stable peptide chain with flexibility [31,32].

### 3.2. Analysis of the Template

**Figure 3:**
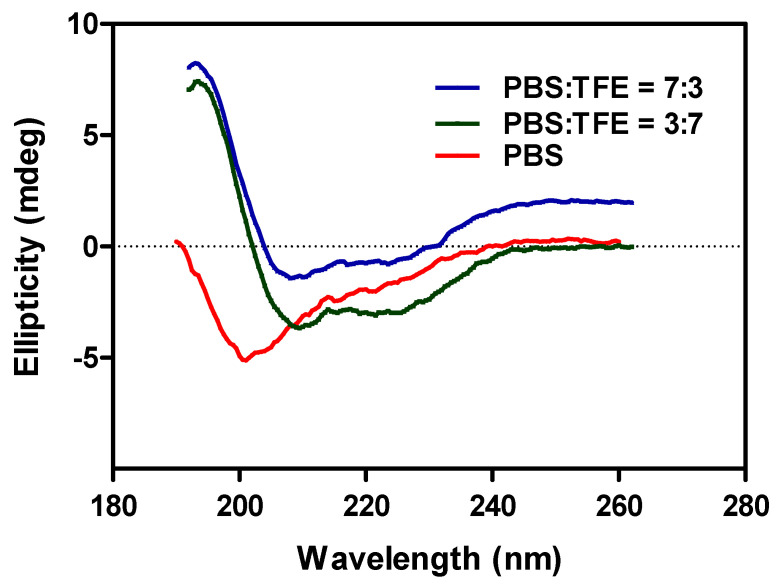
The circular dichroism (CD) spectrum of the PPT ^233–245+G^ segment in different solvent systems.

Furthermore, the template was synthesized using a CEM Discover Microwave Synthesizer (Kohan Co., Taipei, Taiwan) at National Dong Hwa University (Hualien, Taiwan). The peptide segments PPT^107–116^, PPT^145–155^, PPT^169–178^, and PPT^233–245+G^ were selected as templates. Initially, these segments were fabricated using the Fmoc solid-phase peptide synthesis [22]. Further, the purity of the template molecules was confirmed by HPLC equipped with an RP-18 (flow rate- 1 mL/min). Among the selected peptide segments, PPT^107–116^ and PPT^169–178^ showed a purity higher than 96%. Contrarily, the other two segments, PPT^145–155^ and PPT^233–245+G^, had a lower purity of around 88%, which could be attributed to their longer length, leading to a difficulty in the purification of those peptide segments [34]. Further, the molecular mass of the template was analyzed using a Matrix-Assisted Laser Desorption/Ionization-Time of Flight (MALDI/TOF) mass spectrometer (MS) (Bruker, Bremen, Germany) with a matrix consisting of 2,5-dihydroxybenzoic acid (DHB). The reported m/z values of PPT^107–116^, PPT^145–155^, PPT^169–178^, and PPT^233–245+G^ were observed at 1017.69, 1125.56, 1037.39, and 1614.24 [M + Na]^+^, respectively. Further, the peptide segment PPT^233–245+G^ was analyzed with a (JASCO, J-715, Tokyo, Japan) circular dichroism (CD) spectrometer to validate the helix structure in the mixtures of buffer and TFE (Figure 3). Usually, the helical peptides possess negative bands at 208 and 225 nm in a mixture of PBS and TFE, while the peptides with random coil structures show negative bands at 200 nm in PBS [35]. After that analysis, the selected PPT peptide segments were used to generate helix cavities using the PCIMPs-based approach.

### 3.3. Characterization of MPs and PCIMPs

#### 3.3.1. FTIR Analysis

**Figure 4:**
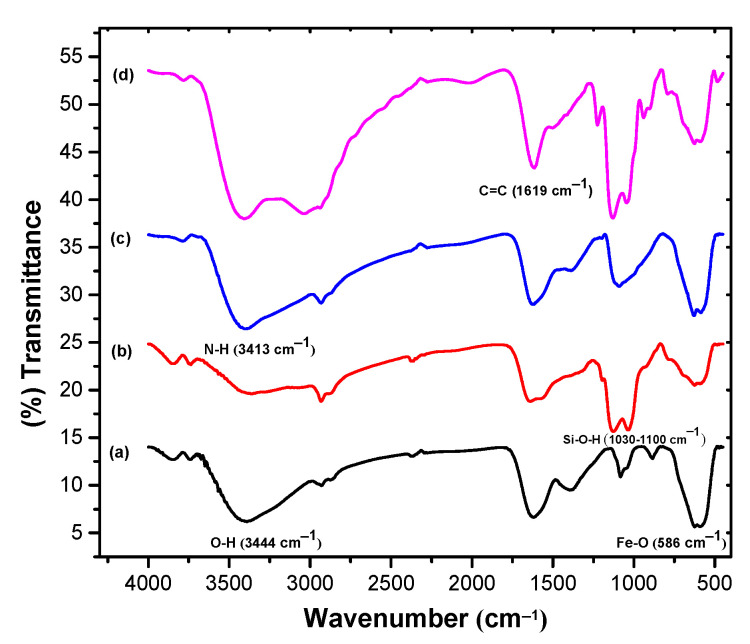
FTIR spectra of (**a**) Fe_3_O_4_, (**b**) Fe_3_O_4_@APTMS, (**c**) Fe_3_O_4_@APTMS-GA, and (**d**) Fe_3_O_4_@APTMS-GA-acrylate.

A Fourier-transform infrared spectrometer (FTIR, Bruker TENSOR 27, Ettlingen, Germany) was employed to examine the successive surface modifications on MPs (Figure 4). The peaks at 586 cm^−1^ and 3444 cm^−1^ can be ascribed to Fe-O stretching vibration and O-H stretching of Fe_3_O_4_ (Figure 4a). The characteristic peaks of silanol groups (Si-O-H) on the surface of Fe_3_O_4_ at 1030 cm^−1^, as well as at 1100 cm^−1^, and the peak at 3421 cm^−1^ can represent the characteristic peaks of NH_2_ (primary amine) of APTMS, indicating the successful modification of the Fe_3_O_4_ nanoparticles surface with amine groups (Figure 4b) [36,37,38]. Additionally, the peaks near of 3413 cm^−1^ can represent the existence of the N-H functional group, and no peak at 1739 cm^−1^ can indicate the C=O group at both ends of the glutaraldehyde molecule reacted with NH_2_, attributed to the established stable secondary structure. The secondary amine-modified iron nanoparticles are more reactive than the primary amine because the inductive effect of secondary amine makes them more stable compared to the primary amine (Figure 4c). The peak at 1619 cm^−1^ can be ascribed to the characteristic peak of C=C, indicating successful acrylation of the MPs (Figure 4d) [39].

#### 3.3.2. FE-SEM Analysis

**Figure 5:**
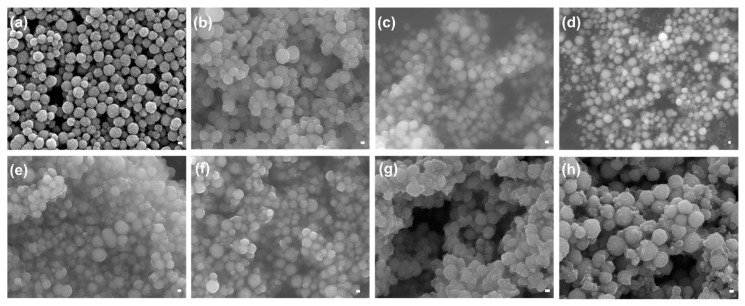
FE-SEM images of (**a**) Fe_3_O_4_, (**b**) Fe_3_O_4_@APTMS, (**c**) Fe_3_O_4_@APTMS-GA, (**d**) Fe_3_O_4_@APTMS-GA-acrylate, (**e**) PCIMPs^107–116^, (**f**) PCIMPs^145–155^, (**g**) PCIMPs^169–178^, and (**h**) PCIMPs^233–245+G^ (Scale bar: 100 nm).

The surface morphology of various MPs and PCIMPs was analyzed using a Field Emission Scanning Electron Microscope (FE-SEM, JEOL JSM-7000F/JEOL Ltd., Tokyo, Japan) (Figure 5). As a result, it was observed that the fabricated Fe_3_O_4_ particles were spherical, showing a uniform size distribution with an average size of ~237 nm (Figure 5a). Further, APTMS immobilization on Fe_3_O_4_ nanoparticles resulted in substantial changes in the size and shape of those MPs, having increased their average size to ~278 nm (Figure 5b). The subsequent immobilization of glutaraldehyde on the MPs resulted in an increase in their average size to ~309 nm (Figure 5c). Notably, a slight aggregation can be observed after the successive surface modification on the MPs. This could be because nanoparticles treated with different solvents and dry samples were collected after the surface modification. The dry power shows strong aggregation, as reported in previous studies [40]. Further, the acrylate monomer conjugation with MPs resulted in an average size of ~323 nm (Figure 5d). Amongst all PCIMPs, the PCIMPs^107–116^, PCIMPs^145–155^, and PCIMPs^169–178^ have shown similar size at ~370 nm, whereas PCIMPs^233–245+G^ were comparatively larger at ~408 nm (Figure 5e–h).

### 3.4. Binding Studies of PCIMPs

For comparison, the binding affinities of the PPT to each PCIMPs were measured by the linear regression curve based on the Scatchard equation. As shown in Table 1, the PCIMPs ^233–245+G^ had the lowest K_d_ value (0.21 μM) of all the PCIMPs. It was observed from the results that the K_d_ values showed a decreasing trend with an increase in the number of peptide residues. Therefore, the higher the number of peptide segments in the template, the better the observed binding affinities. For instance, for the 14-mer peptide, the K_d_ was 0.21 μM, and it showed better affinity when compared to the 10 and 11-mer peptides [19]. Similarly, for PCIMPs^145–155^, the K_d_ value was 0.38 μM, and it presented a better affinity than that of a 10-mer peptide. On the other hand, both PCIMPs^107–116^ and PCIMPs^169–178^ have shown a similar number of peptide residues in the template. In this case, affinities of the PPT to PCIMPs were more closely related to the molecular weight of the template residues. For example, the K_d_ value of the PCIMPs^169–178^ was 0.55 μM, which showed a better binding affinity than PCIMPs^107–116^ (0.65 μM).

Previously, Griffete and colleagues developed a magnetic-protein imprinted polymer (M-PIP) by combining photopolymerization with a grafting approach onto surface-functionalized MPs. The authors demonstrated that the green fluorescent proteins were bound to MIPs in less than 2 h with a high affinity (K_d_ = 0.29 μM) [41]. In another study, MIPs were synthesized using a solid-phase approach on metal chelate functionalized glass-beads to immobilize trypsin using its surface histidine. Although less cross-reactivity with other proteins was observed, the dissociation constant value of the MIP-trypsin complex was 0.237 μM [42], with a lagging binding capacity. Notably, in this study, the PCIs developed on the surface of MPs create recognition sites that are complementary to the protein conformational structure and, therefore, significantly increase the specificity toward the targeted protein. The best binding performance of PCIMPs^233–245+G^ occurred in 10 min with a high affinity (K_d_ = 0.21 μM). Upon a comprehensive evaluation of binding affinities and absorption time, it was apparent that conformational imprints on MPs acquired better results in these protein-imprinted particles. Together, our findings indicated a higher affinity of protein (PPT) to PCIMPs ^233–245+G^ (K_d_ = 0.21 μM), in comparison to the other MIPs grafting methods.

### 3.5. Kinetic Parameters of PPT and PPT/PCIMPs

**Figure 6:**
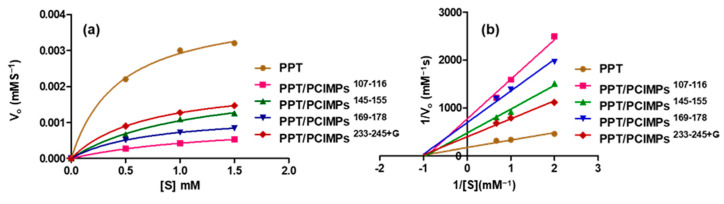
(**a**) Michaelis-Menten and (**b**) Lineweaver-Burk plots of PPT and PPT/PCIMPs obtained with various *N*-benzoyl-L-arginine ethyl ester (BAEE) solutions (1.5, 1, 0.5 mM). Note: The standard deviation for the Michaelis-Menten kinetics plot of PPT in different concentration (1.5 mM to 0.5 mM) is 1 × 10^–4^ whereas and PPT/PCIMPs ^233–245+G^ is 1.41067 × 10^–5^, 7.2111 × 10^–6^, and 5.50757 × 10^–6^ for (1.5, 1, and 0.5 mM).

In addition, the PCIMPs bound to PPT exhibited excellent catalytic activity. To demonstrate this aspects, the kinetic parameters of PPT and PPT/PCIMPs were explored by varying the BAEE substrate concentration (0.5–1.5 mM). They were then calculated using the Michaelis-Menten plot (Figure 6a). As shown in Table 2, among all the PPT/PCIMPs, PPT/PCIMPs ^233–245+G^ had the best kinetic parameters. The *K_m_* value of PPT (0.36 mM) was almost similar to that of the PPT/PCIMPs ^233–245+G^ (0.42 mM), which could be due to the high feasibility of forming an enzyme-substrate complex, and also a lower diffusion restraint imposed on the flow of the substrate and product molecules from the grafted polymer matrix of the MPs [43,44]. The *V_max_* values were found to be 3.2 × 10^–3^ mMs^−1^ and 1.47 × 10^–3^ mMs^−1^ for PPT and PPT/PCIMPs ^233–245+G^, in which the *V_max_* was decreased for PPT/PCIMPs when compared to the free enzyme. The plausible reason might be due to the created steric hindrances that restrict the substrates’ transport, enhance diffusional creation limitations, and decrease the enzyme’s catalytic properties. These conclusions are in agreement with the results reported literature [45,46].

Respectively, it was observed that the *k_cat_* value of PPT/PCIMPs^233–245+G^ was lower than that of PPT. The decrease of *k_cat_* values upon immobilization of enzymes are frequently reported [13,47,48]. These findings suggest a limited diffusion of the substrate to the active site and higher structural rigidity of the immobilized PPT. Our results are quite comparable and in agreement with the ones reported in the literature [47,48,49,50]. Furthermore, the trypsin inhibition by PCIMPs was investigated by performing enzyme assays in the Tris-HCl buffer at pH 6.2, using BAEE as the substrate at various concentrations. The Lineweaver Burk plot (1/V_o_ versus 1/S) is as shown in Figure 6b. It reveals that the PCIMPs exhibited non-competitive inhibition towards trypsin, in which the PCIMPs acted as inhibitors, while the BAEE functioned as a substrate. In non-competitive inhibition, the respective inhibitors bind to the free enzyme and the enzyme-substrate complex with the same affinity. Further, the inhibitor reduces the activity of the enzyme and binds equally well to the substrate [51,52].

### 3.6. Reusability

**Figure 7:**
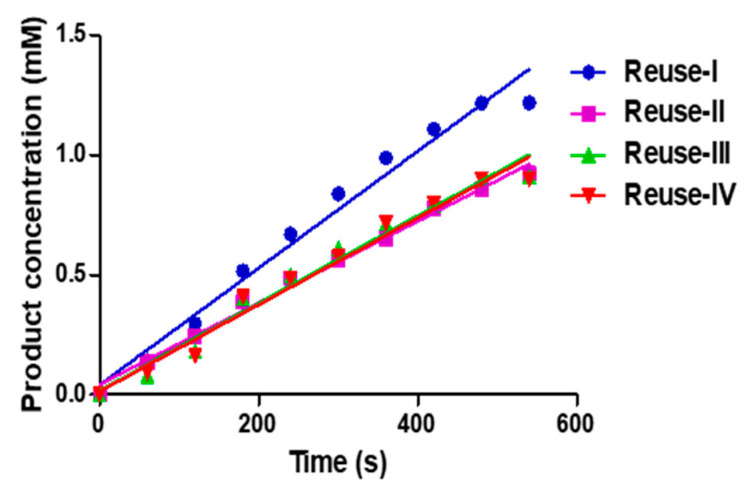
Reusability of PPT/PCIMPs^145–155^.

Additionally, the reusability of PPT/PCIMPs was examined. Initially, 10 mg of PPT/PCIMPs ^145–155^ was added to 8.8 mL of a 1.5 mM BAEE solution (50 mM Tris-HCl buffer, pH 7.6). The product concentration was monitored using HPLC. The test was conducted consecutively four times. It was observed from the results that the PPT/PCIMPs retained 90% of activity in 540 sec in the first cycle; however, in the subsequent cycles, it slightly dropped. The activity of the protein sustained after four cycles is as shown in Figure 7.

### 3.7. Comparison Studies of the Proposed PPT/PCIMPs with Other Methods

The catalytic hydrolysis performance of the fabricated PPT/PCIMPs was compared to previous studies (Table 3). For example, Atacan and colleagues modified the surface of Fe_3_O_4_ nanoparticles with gallic acid. According to their research, *K_m_* values of trypsin and immobilized trypsin were 5.1 and 7.88 mM, respectively, indicating that the immobilized trypsin has less affinity for the substrate, which might be attributed to the loss of enzyme flexibility. Although immobilized trypsin retained 92% of its initial activity after four months of storage at 4 °C, there was a dramatic decrease in its activity after being reused eight consecutive times [49]. In another study, trypsin was immobilized on polymer and grafted magnetic beads, in which the *K_m_* for immobilized trypsin was found to be 13.6 mM, 1.4-fold higher than free trypsin, while *V_max_* value was found to be 3946 U/mg, 1.5-fold lower than for the free trypsin, indicating that a change in the affinity of the enzyme towards the substrate occurred upon its immobilization [50]. In different work, by Bayramoglu and colleagues, polymer grafted magnetic beads were activated with glutaraldehyde for the immobilization of trypsin on affinity ligands attached to the beads’ surface. Moreover, the reusability and activity were relatively good in this study when compared to the above work. The *K_m_* and *V_max_* values obtained for the immobilized trypsin were of 16.8 mM and 5115 U/mg, 1.8-fold higher and 1.5-fold lower than free trypsin, respectively. The *K_m_* values could be explained by the fact that there existed conformational changes during enzyme immobilization [53].

Upon a comprehensive evaluation of kinetic parameters, it was evident that the elegant helical cavities imprinting strategy created recognition sites on the MPs surface, in which the enzymes were tightly bound. Moreover, it was achieved an improved catalytic hydrolysis in comparison to other previous studies. The best performance of PPT/PCIMPs for hydrolysis of BAEE had the following values for the kinetic parameters *K_m_*, *V_max_*, and *k_cat_* values were 0.42 mM, 1.4 μM·s^−1^, and 1.16·s^−1^. Additionally, PPT/PCIMPs-imprinted materials exhibited stable catalytic activity and reusability.

## 4. Conclusions

In conclusion, a state-of-the-art method for point immobilization of enzymes on magnetic particles is accomplished. To maintain the catalytically competent state of an enzyme, an immobilized enzyme at a maximum degree of freedom is the ultimate choice. Our systems operate by binding enzyme partially and maintaining the remaining part of the enzyme free. The combination of site fixation with the use of conformation-specific PCIMPs could boost the catalytic process in many enzymes. Moreover, the experimental results also indicated the inhibition effect on capturing at the α-helix region to interfere with catalysis flexibility. The *K_m_* of PPT/PCIMPs^233–245+G^ was slightly higher than that of PPT, resulting in lower diffusion limitations of the substrate and product molecules from the polymer matrix to forming an enzyme-substrate complex. Consequently, this method is an appropriate choice for realizing the relationship between each segment’s flexibility and catalytic activity. We thus believe the PCIMPs strategy can be more widely applied in green chemistry as a nano biocatalyst.

## Figures and Tables

**Table 1:** Binding affinity values of various PCIs on magnetic particles (PCIMPs) to PPT.

MPs	PCIMPs^107–116^	PCIMPs^145–155^	PCIMPs^169–178^	PCIMPs^233–245+G^
Residue	10	11	10	14
[K_d_] μM	0.65	0.38	0.55	0.21
[B_max_] nM	0.75	1.11	0.95	1.11
B_max_/K_d_	1.15	2.92	1.73	5.29

**Table 2:** Kinetic parameters obtained from the Michaelis-Menten plot.

MPs	PPT	PPT/PCIMPs^107–116^	PPT/PCIMPs^145–155^	PPT/PCIMPs^169–178^	PPT/PCIMPs^233–245+G^
*V_max_* (mM s^−1^)	3.2 × 10^−3^	0.53 × 10^−3^	1.25 × 10^−3^	0.84 × 10^−3^	1.47 × 10^−3^
[*K_m_*] mM	0.36	0.52	0.46	0.44	0.42
*k_cat_* (s^−1^)	2.6	0.62	0.99	0.78	1.16
*k_cat_*/*K_m_* (mM^−1^ s^−1^)	7.32	1.19	2.15	1.77	2.79

Note: PPT= porcine pancreatic alpha-trypsin, PPT/PCIMPs = immobilized PPT.

**Table 3:** Comparison studies of proposed PPT/PCIMPs with other methods.

Trypsin/Immobilized Trypsin	*K_m_*	*V_max_*	*k_cat_* (s^−1^)	*k_cat_*/*K_m_* (mM^−1^ s^−1^)	Reference
BPT/Immobilized BPT	5.1/7.88 mM	23/18.3 mM min^−1^	-	-	[42]
BPT/Immobilized BPT	9.7/13.6 mM	5890/3946 U/mg	-	607/290	[43]
BPT/Immobilized BPT	9.3/16.8 mM	7345/5115 U/mg	-	-	[44]
PPT and PPT/PCIMPs^233–245+G^	0.36/0.42 mM	3.2 × 10^−3^/1.47 × 10^−3^ mM s^−1^	2.6/>1.16	7.32/2.79	This study

Abbreviations: Bovine Pancreas Trypsin (BPT), Porcine Pancreatic Trypsin (PPT), Note: U is defined as μmol.

## Data Availability

Not applicable.
